# Direct observation of polar tweed in LaAlO_3_

**DOI:** 10.1038/srep27193

**Published:** 2016-06-02

**Authors:** Ekhard K. H. Salje, Marin Alexe, Sergey Kustov, Mads C. Weber, Jason Schiemer, Guillaume F. Nataf, Jens Kreisel

**Affiliations:** 1 Materials Research and Technology Department, Luxembourg Institute of Science and Technology, 41 Rue du Brill, L-4422 Belvaux, Luxembourg; 2 Department of Earth Sciences, University of Cambridge, Cambridge CB2 3EQ, UK; 3 University of Warwick, Department of Physics, Coventry CV4 7AL, W Midlands, England; 4 Universite des Illes Balears, Department Fisica, E-07122 Palma De Mallorca, Spain; 5 Physics and Materials Science Research Unit, University of Luxembourg, 41 Rue du Brill, L-4422 Belvaux, Luxembourg; 6 SPEC, CEA, CNRS, Université Paris-Saclay, CEA Saclay, 91191 Gif-sur-Yvette Cedex, France

## Abstract

Polar tweed was discovered in mechanically stressed LaAlO_3_. Local patches of strained material (diameter ca. 5 μm) form interwoven patterns seen in birefringence images, Piezo-Force Microscopy (PFM) and Resonant Piezoelectric Spectroscopy (RPS). PFM and RPS observations prove unequivocally that electrical polarity exists inside the tweed patterns of LaAlO_3_. The local piezoelectric effect varies greatly within the tweed patterns and reaches magnitudes similar to quartz. The patterns were mapped by the shift of the *E*_*g*_ soft-mode frequency by Raman spectroscopy.

High memory capacities and electrical wiring on a much finer scale than achievable with current technologies may be possible when active elements in devices are not related to bulk properties but when only domain boundaries contain the desired functionalities^1^,^2^,^3^,^4^,^5^,^6^,^7^,^8^,^9^,^10^,^11^,^12^. Much work was dedicated to exploring highly conducting domain walls as a replacement of wires in device applications. Such domain boundaries are designed to carry high currents and it was the discovery of superconducting twin boundaries^13^ that opened a wide field of applications in ‘domain boundary engineering’ where the domain boundary is the device and were the design of the device materials depends largely on tailoring appropriate domain boundaries^1^,^2^. Furthermore, electric dipole moments were observed inside ferroelastic domain walls so that switchable ferroelectricity is confined to domain walls and cannot interfere with depolarization fields and additional switching of domains in the bulk. The length scale of the active device was then restricted to the size of domain walls or to even smaller structures such as Bloch walls inside domain walls^13^,^14^,^15^,^16^,^17^,^18^,^19^. This approach requires – at least at the present sensitivity for the detection of ferroic functionalities – that many walls cooperate to induce a measurable macroscopic response to applied fields.

The aim is hence to produce high wall concentrations. The highest concentration was predicted for a tweed structure, which is a densely interwoven network of domain walls^20^,^21^,^22^,^23^,^24^,^25^. Tweed has another property: it will form a domain glass with a non-ergodic response to external forcing. Domain glass^26^,^27^ is akin to polar nano-regions, which are known to exist in relaxor materials^28^,^29^,^30^. Lloveras *et al.*^31^ have argued that spatially heterogeneous states like tweed depend crucially on the elastic anisotropy while detailed stability simulations showed that tweed structures are omnipresent in any ferroelastic precursor pattern. These arguments indicate that tweed is stabilized by defects while dynamic tweed^23^ exists also for very low defect concentrations^32^. It was then argued that tweed structures are polar, either via the flexoelectric effect or via bi-linear coupling between the strain and local dipole moments^33^,^34^,^35^,^36^,^37^ although such polarity has never been seen. Here we report the first experimental evidence for piezoelectricity of a tweed structure where the uniform parent structure is centrosymmetic and shows no polarity.

Over the last decade, a general search for tweed structures in systems with low defect concentrations has made little progress. Several attempts failed to produce tweed by cold-shearing SrTiO_3_^32^,^38^. The main obstacle to the discovery of tweed is the high mobility of tweed patterns, which remain invisible optically or by transmission electron microscopy. Nevertheless, diffraction evidence was found both in alloys and ceramics^23^. A prime candidate for tweed is LaAlO_3_, which is ferroelastic^39^ and contains a high density of mobile twin walls^40^. Wall polarity was never seen in LaAlO_3_ in contrast to CaTiO_3_ and SrTiO_3_^10,14^ where the local dipoles are related to the off-centering of Ti inside an octahedral oxygen cage. LaAlO_3_ has no known ferroelectric instability and wall polarity was hereto unknown for perovskites structures with Al in octahedral position. Nevertheless, very weak piezoelectricity was previously suspected in some samples^39^ (but never confirmed by diffraction based symmetry analysis). In this paper, we report a significant piezoelectricity in tweeded LaAlO_3_ samples with low defect concentrations.

It is likely, therefore, that our observation can be generalised to other compounds and leads credence to the initial hypothesis that most (or perhaps all) tweed structures involving anion and cation lattices are polar.

## Sample Characterisation

A 2-inch-disc of LaAlO_3_ (MTI Corporation, USA) was analysed by microprobe analysis. The sample was slightly more defect-rich than typical LaAlO_3_ specimen: the purity was 99.98% rather than 99.99% as commonly seen in other samples (e.g. Crystal GmbH, Berlin, Germany). The chemical composition is listed in Table 1, the main impurities are Cl and Si.

**Figure 1:**
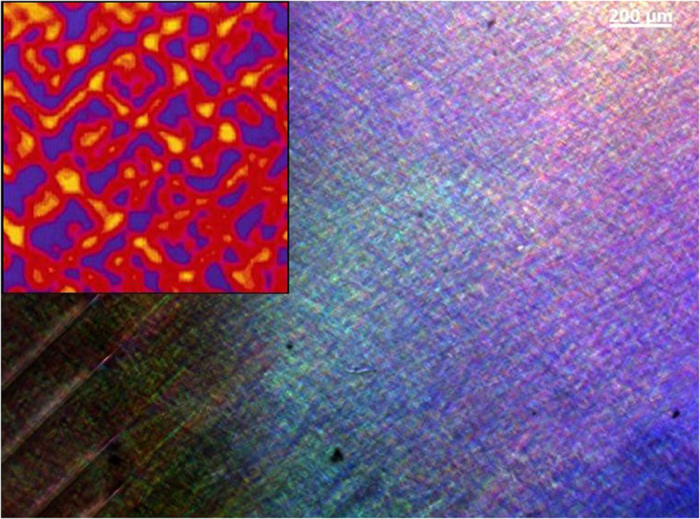
Microscopic image of a cut slice of LaAlO_3_. Needle domains are visible at the left side of the image. A tweed microstructure is seemed throughout the sample. The inset shows a simulation of a tweed structure of a size of 100 unit cells taken from^41^.

The disc was cut into narrow stripes of 1 cm width. The as-grown sample was optically free of tweed at room temperature and contained a small number of needle twins. The cutting was performed with a diamond saw (Buehler). The cutting induced stress fields in the sample. As a consequence, additional needle domains were induced at the edge of the sample and the entire sample assumed an almost uniform, coarse-grained tweed microstructure. A typical optical image of the sample after cutting is shown in Fig. 1. A tweed microstructure is seemed throughout the sample. The inset shows a map of strain order parameter of a tweed structure obtained by Monte-Carlo simulation (reproduced from ref. 41).

## Results

**Figure 2:**
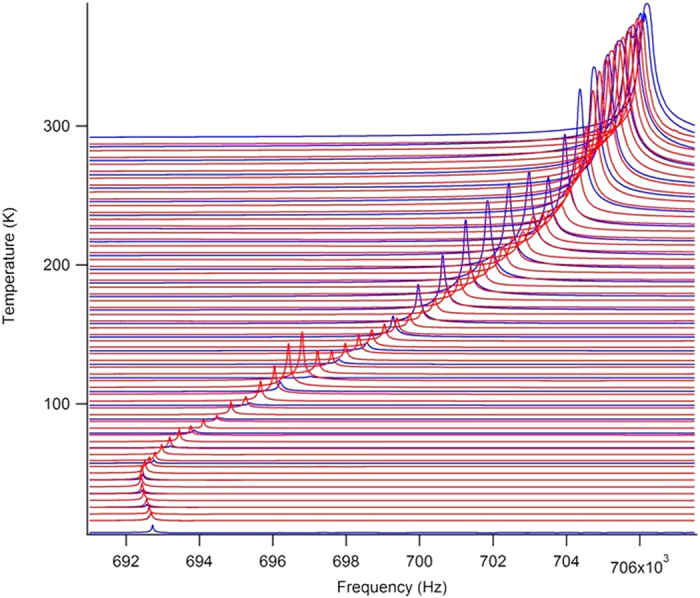
RPS spectra of LaAlO_3_ below room temperature. Blue curves are for cooling and red curves for heating experiments. The amplitude of the signal is similar to that of agate^47^.

Weak electric fields applied at frequencies between 100 kHz and 10 MHz excite strong piezoelectric vibrations in LaAlO_3_ with a tweed structure but not in uniform samples. Amongst the large number of resonance peaks we selected the one with the lowest peak overlap (Fig. 2). This RPS signal is comparable with that of randomized quartz in agate^42^ but is weaker than in tetragonal BaTiO_3_^24^. The observation of RPS signals already proves unequivocally that samples with tweed structures are piezoelectric. Considering the diffraction based point group symmetry 3 m of LaAlO_3_ we find that this piezoelectric point group symmetry is also polar.

**Figure 3:**
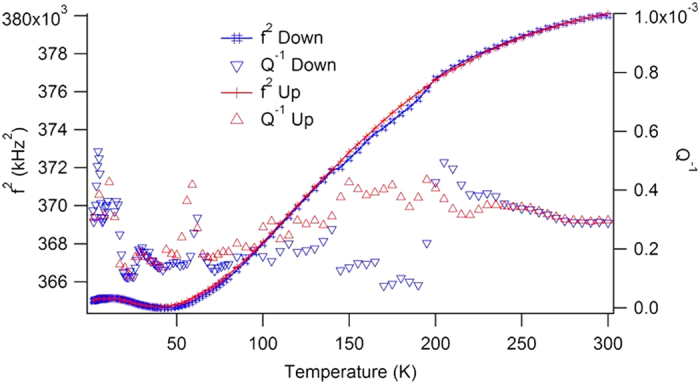
RUS temperature evolution of a RUS resonance below room temperature. The temperature dependences of the resonance signals in RPS and RUS are identical. The damping Q^−1^ is very small showing the excellent quality of the sample and the lack of major pinning centres for twin boundary movements.

The Piezoelectric Resonance Spectroscopy, RPS, method is described in more detail under ‘experimental methods’. We now discuss some details of the RPS observations. The validity of the RPS observation is guaranteed because the peak frequency and its temperature evolution are identical to those of purely mechanical resonances shown in Fig. 3. The temperature evolution of the resonance frequency is unusual, however, as it shows a significant softening on cooling below room temperature. This softening is identical in samples with and without tweed and was already reported in^43^. A first tentative explanation related the softening to a Debye-like dissipation peak, which occurs near 250 K (activation energy 43 ± 6 kJ mol^−1^). The mechanism for this activation process is associated with the modulus *C*_44_. The physical origin of the process in not known. The softening in the temperature interval between 220 K and 70 K is similar to those observed in incipient ferroelastics or ferroelectrics. The softening interval ends with a further dissipation peak at <40 K, its origin was discussed in^27^ in terms of freezing of atomic motions of La and/or Al. LaAlO_3_ thus shows evidence for an incipient structural instability at low temperatures which is potentially analogous to SrTiO_3_^18^. The softening is the same in crystals with and without the tweed structure^27^ and is hence not be related to tweed on a micron-scale. It is possible, however, that tweed on a much finer, submicroscopic scale may exist in most samples^27^. The dynamic excitations in tweed^26^,^27^ are typically low energy phason modes, which strongly reduce the mechanical shear resonance frequencies. Their appearance could explain the observed temperature dependence of LaAlO_3_. The peak at <40 K would then be due to ‘domain’ freezing of the tweed structure. The damping at low temperatures is below 5 10^−4^. It shows the excellent quality of the LaAlO_3_ sample. No Snoek-type relaxations occur. Domain boundary movements may exist but their energy loss is equally extremely small.

**Figure 4:**
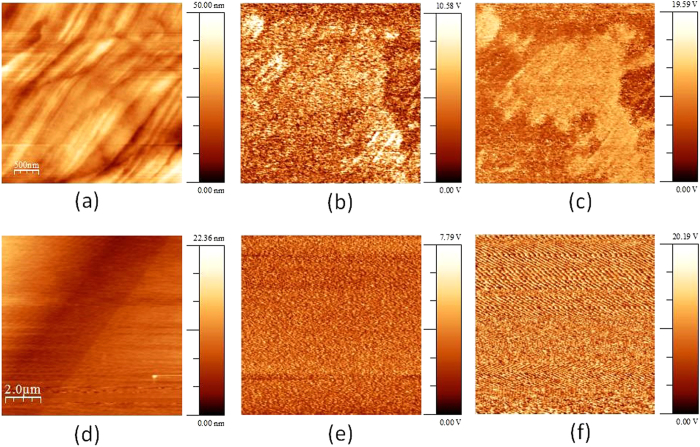
Local piezoelectric activity measured by piezo-force microscopy (PFM). Data of a highly twinned LaAlO_3_ region (**a**–**c**) and an un-twinned LaAlO_3_ region crystal (**d**–**f**). (**a**,**d**) Represent the topography, (**b**,**e**) is the PFM out-of-plane amplitude and (**c**,**f**) is the corresponding phase.

The discovery of polarity by RPS was confirmed by PFM (Fig. 4). The pattern in Fig. 4b,c shows patches of polarity in the (100) plane. The diameters of these patches are around 5 μm. They occur only in part of the sample where tweed was found by optical microscopy (Fig. 4a–c). An un-twinned region of the crystal without microscopically visible tweed showed only background PFM noise (Fig. 4d–f), which corresponds to an effective piezoelectric coefficient of about 1 pm/V while the tweeded sample shows patches of higher and lower signals. The low signal is only 20–30% higher than the background noise while the corresponding effective piezoelectric coefficient in the high signal regions can be as high as (2.6+/−0.2) pm/V. The local piezoelectric coefficient is hence similar to quartz, in agreement with RPS results.

**Figure 5:**
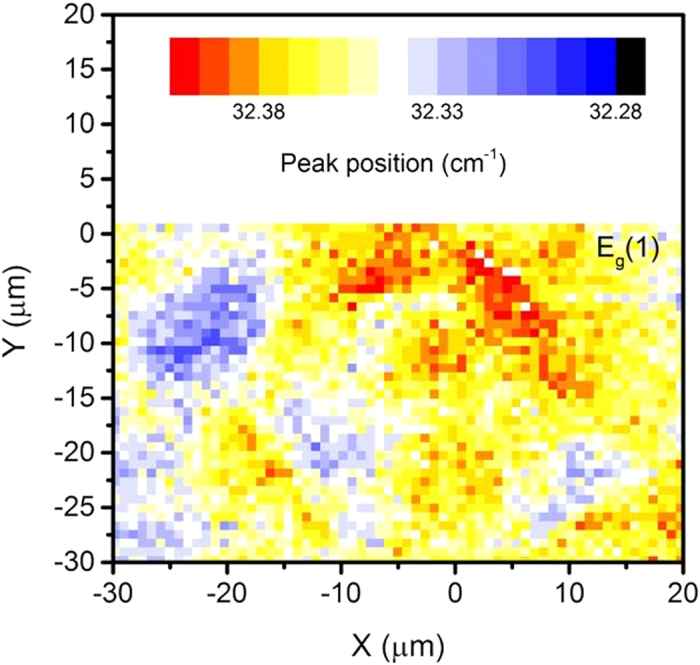
Mapping of a tweed region by the shift of the low-frequency Raman-active *E*_*g*_ mode.

The tweed is observable by Raman spectroscopy using the spatial distribution of peak shifts in a sample with optically visible tweed^39^,^44^ (Fig. 5). The lowest-lying *E*_*g*_ soft mode showed a frequency shift of ca. 0.1 cm^−1^. The peak shift of 0.1 cm^−1^ is correlated with the dominant structural change during the phase transition, namely the rotation of the AlO_6_ octahedra around the trigonal axis. Using the correlation between the octahedral rotation and the shift of the Raman frequencies in ref. 39 leads to the calibration of the maximum local rotation as 1.3 × 10^−3^ degrees. The equivalent temperature shift of the rotation corresponds to 1 K^39^. The tweed pattern may hence be envisaged as a structural fluctuation, which is equivalent to an approximate local temperature fluctuation of 1 K. PFM and Raman signals see similar patterns (Figs 4 and 5), both measurements were performed in reflection mode and emphasize the surface effect. In contrast, the optical image in Fig. 1 was measured in transmission mode and superimposes tweed of several parts of the sample.

## Discussion

Polar tweed is expected to require some additional structural instability which, at first glance, seem not to exist in LaAlO_3_^20^. Nevertheless, some anomalies have been reported which may point to a ‘hidden’ instability. Let us start with the traditional interpretation of the Pm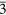 m/R
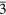
c phase transition in LaAlO_3_ at T_c_ = 813 K which is traditionally approximated by the rotation of centro-symmetric AlO_6_ octahedra around of the pseudocubic [111] axes. The maximum rotation angle at absolute zero temperature is 5.6°^39^. No evidence by x-ray or neutron diffraction was found previously that the R
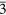
c symmetry is lowered to a non-centrosymmetric space group. Nevertheless, several aspects of the phase transition are incompletely described by this octahedra-rotation model. The order parameter of the transition involves a large deformation of the AlO_6_ octahedron and, possibly, additional deformations of the 12-fold coordinated La site. Only the full thermodynamic order parameter shows a second-order Landau transition near T_c_. According to Howard **et al.**^45^ AlO_6_ octahedra in LaAlO_3_ suffer a slight compression between triangular faces aligned perpendicular to [111] of the cubic parent structure and a slight expansion in the plane perpendicular to this. The following observations indicate structural instabilities beyond the octahedral tilt model:

1.  The rhombohedral spontaneous strain and the local rotation angle for LaAlO_3_ do not extrapolate to the same transition temperature and show different temperature dependences. The spontaneous strain disappears at 830 K while the rotation angle shows additional anomalies near 730 K^39^.
2. The temperature evolutions of the two soft mode frequencies (*A*_*1g*_ and *E*_*g*_) are not proportional to each other at T < 730 K, and the spontaneous strain is not proportional to the square of the AlO_6_ rotation angle. These anomalies are formally consistent with biquadratic coupling between the primary order parameter of the transition^39^ and a second, unknown process. From dielectric measurements, which indicate a smooth but rapid increase in conductivity in the temperature range 500–800 K, this second process may be related to hopping of intrinsic oxygen vacancies and possible local lattice distortions. Furthermore, twin domains are mobile above 730 K but are frozen below 730 K^46^, which may also be related to defects including oxygen vacancies. The measured specific heat anomaly peaks at 813 K^39^, which is below the extrapolated T_c_ of the octahedral deformation at 830 K.
3. The decrease of c_44_ under cooling below room temperature is not mirrored by an increase of the dielectric response, which excludes any mechanism involving an incipient ferroelectric transition^39^,^43^. In the equivalent situation of the incipient ferroelectric transition in KTaO_3_^47^ a steep increase of the dielectric susceptibility indicates the potential nucleation of a ferroelectric phase at low temperatures. Similarly results were found for SrTiO_3_^48^. The elastic softening of LaAlO_3_ cannot be related to such an incipient ferroelectric phase but to an incipient ferroelastic transition^43^. Its intrinsic lattice instability is unknown and may play a major role in the formation of the tweed pattern. A space group C2/m was discussed in^43^.
4.  The order parameter saturation in the quantum regime^49^ is different for the octahedral rotation and the octahedral deformation. The octahedral rotation saturates at 260 K while the octahedral distortion saturates at 150 K. The split of these two saturation temperatures is highly unusual in perovskite structures and suggests non-linear rotation-translation coupling. The low temperature structure is characterized by increasing octahedral distortions while their rotation angles remain almost constant under cooling.
5. Dipolar pattern formation was anticipated from dielectric resonator measurements of the loss tangent tanδ and relative permittivity ε_r_ at low temperatures and 4–12 GHz^50^. A variety of single crystals grown by different techniques were investigated. The loss tangent tanδ is largely sample independent and shows a linear frequency dependence and monotonous temperature variation from 8 × 10^−6^ at 300 K to 2.5 × 10^−6^ at 150 K and 4.1 GHz at T > 150 K. The loss tangent below 150 K is characterised by a peak at ca. 70 K. The height of this peak is frequency and sample dependent. The peak was explained by defect dipole relaxations. The activation energy of the relaxation process is 31 meV. This low value was taken as evidence that the defect dipoles are associated with interstitials, possibly impurities in interstitial positions^50^. This model can be reconciled with our polar tweed patterns if local strain is sufficient to generate defects or correlate defects to follow the strain deformation.
6. The entropy of the Pm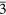 m-R
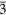
c phase transition is larger than normal for an octahedra-tilt transition. The ‘a’ coefficient of the Landau potential of the cubic ↔ tetragonal transition in SrTiO_3_ is 0.65 J mol^−1^ 51,52 giving a total excess entropy at order parameter Q = 1 of ~0.33 J mol^−1^ K^−1^. For LaAlO_3_ ‘a’ is 3.9 J mol^−1^ K^−1^ and the equivalent total excess entropy is ~1.95 J mol^−1^ K^−1^. This large value suggests some contribution from configurational effects such as the displacement of Al and La, which could lead to polarity of the AlO_6_ and LaO_12_ groups.
7. Sathe and Dubey^53^ claim a weak additional peak in Raman spectra which displayed increasing intensity below ~240 K. They associated this peak with other weak anomalies at higher temperatures and considered the possibility that the local symmetry could be R3c or R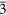, again due to displacements of La and Al from their high symmetry positions in the R
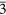
c structure.

These seven arguments show that the structural state of LaAlO_3_ below T_c_ is not simply defined by the octahedral tilt and that other atomic movements exist. If these movements are strain related we would expect that the maximum strain contrast in the tweed is equivalent to ca. 1 K-temperature variation in the structural state in^39^. This strain contrast is 2.4 × 10^−6^ for e_1_ and 3 × 10^−6^ for e_4_. The spatial gradient extends over some microns so that a simple flexoelectric effect may be too small to explain the observed polarity of the tweed pattern^54^. Structural instabilities related to the polar off-centering of Al and possibly La can explain the effect. A similar situation was found in tweeded BaTiO_3_ where Ti at T ≫ T_c_ is dynamically disordered over off-centered octahedral sites on fast time scales^55^,^56^.

We finally mention that polarity in thin films of LaAlO_3_ have been reported in the pioneering paper by Sharma *et al.*^57^. These authors describe the switchable hysteretic electro-mechanical behaviour of crystalline epitaxial LaAlO_3_ thin films associated with polarization induced by electrical and mechanical fields. They suggest that the ferroelectric-like response of the thin films is mediated by the field-induced ion migration in the bulk of the film, which could indeed also play a role in surface near regions in bulk samples.

## Conclusion

We have proven that polar tweed structures exist. Similar observations in ferroelectric materials in their paraelectric phase may simply be related to some local short range order. However, as LaAlO_3_ is not ferroelectric and has no incipient ferroelectric instability we have shown that polar tweed exist even in purely ferroelastic materials. This result may possibly be generalized: (almost) all ferroelastic perovskite materials may be polar in their tweed state. If this hypothesis is true, we may ask why has such polar tweed not been observed before? As we show in this paper, the amplitude of polarity is very small in LaAlO_3_ and the effect may simply have been missed in other materials. Furthermore, not all perovskites form tweed easily and it may take a specific effort to generate tweed. Nevertheless, once the existence of polar tweed in non-polar LaAlO_3_ is known, it may open avenues to the discovery of polar tweed structures in other materials.

Our findings may be important also for LaAlO_3_ substrates. We cannot exclude that such substrates contain polar tweed in their surface layers when mechanically worked (e.g. by cutting). These substrates will then interact with deposited thin films not only by shear deformations but also by polar interactions which may dominate when the thin film is ferroelectric. In particular ultrathin ferroelectric films may reflect the polarity of the underlying substrate and show, equally, tweed like features.

## Experimental Methods

Resonant Piezoelectric Spectroscopy, RPS, shows the polarity of the structures. The experimental arrangement is based on the excitation of elastic waves via piezoelectric coupling inherent to the sample. A small AC voltage (1–20 V) is applied across the sample, which is balanced across its corners or parallel faces between the ends of two piezoelectric transducers. The driving voltage leads to the excitation of local distortions that, when collective, lead to macroscopic resonant elastic waves. Great care is taken to disallow cross-talk between the applied field and the mechanical detectors. Additionally, each experiment was performed with uniform and tweed samples. The uniform samples never showed an RPS signal but all tweed samples did. The sample size for the final experiment was 5 × 5 × 1 mm. Any mechanical resonance is transmitted from the sample to the receiver transducer attached to the sample inside a He-cryostat, similar to Resonant Ultrasound Spectroscopy (RUS)^58^,^59^,^60^.

The difference between RPS and Resonant Ultrasonic Spectroscopy, RUS, relates to the excitation of the waves: RPS uses the sample itself as an emitter while in RUS the waves are excited mechanically by an emitter transducer. Switching from RPS to RUS is achieved by applying the AC voltage across the emitter transducer rather than across the sample^24^.

AFM studies were performed using a commercial AFM XE-100, Park Systems working in contact mode. Piezo-response and vertical and lateral piezoresponse force microscopy (PFM) images were routinely obtained with an AC voltage of 5 Vrms at 22.5 kHz applied to a Pt coated silicon cantilever with a spring constant of 2.8 N/m (NSC14, μMasch). Local piezoelectric coefficient has been estimated from the slope of the PFM signal versus the ac excitation signal and by comparing the slopes obtained using the same cantilever for the investigated LaAlO_3_ samples, a PZT 20/80 epitaxial film and a x-cut quartz crystal.

Raman spectra were collected with a Renishaw in Via Reflex Raman Microscope using an excitation wavelength of 633 nm with a spectral cut-off at 10 cm^−1^ and a spectral resolution of 0.4 cm^−1^. Measurements were performed in micro-Raman mode with an objective with numerical aperture 0.75 providing a theoretical laser spot size of 1 μm. Mapping experiments were conducted with a step size of 0.8 μm. The sample was in a thermally stable environment, the time for a complete measurement was 48 hours.

## Additional Information

**How to cite this article**: Salje, E. K. H. *et al.* Direct observation of polar tweed in LaAlO_3_. *Sci. Rep.* **6**, 27193; doi: 10.1038/srep27193 (2016).

## Figures and Tables

**Table 1:** Chemical impurities measured in a tweed sample of LaAlO_3_.

Fe	1.53 ppm	Pb	7.5 ppm
Na	0.02 ppm	Pr	7.5 ppm
K	0.03 ppm	Sm	7.5 ppm
Zn	0.04 ppm	P	0.02 ppm
Si	34.08 ppm	Ti	0.2 ppm
Cl	76.02 ppm	Ni	0.02 ppm
Ca	10.67 ppm	Ga	0.03 ppm
Cu	1.54 ppm	Nd	7.5 ppm
